# A Systematic Review on Sexual Health and Drug Use Prevention Interventions for Black Girls

**DOI:** 10.3390/ijerph19063176

**Published:** 2022-03-08

**Authors:** Ijeoma Opara, Kimberly Pierre, Maame Araba Assan, Laurel Scheinfeld, Courtnae Alves, Kristina Cross, Ashley Lizarraga, Bridgette Brawner

**Affiliations:** 1 Department of Social & Behavioral Sciences, Yale School of Public Health, Yale University, New Haven, CT 06520, USA; 2 Center for Interdisciplinary Research on AIDS, Yale School of Public Health, Yale University, New Haven, CT 06520, USA; 3 Irvington Department of Health and Senior Services, Irvington, NJ 07111, USA; kpierre@irvingtonnj.org; 4 Public Health Management Corporation, Philadelphia, PA 19102, USA; araba125@gmail.com; 5 Health Sciences Library, Stony Brook University, Stony Brook, NY 11794, USA; laurel.scheinfeld@stonybrook.edu; 6 School of Health Technology and Management Health Science, Stony Brook University, Stony Brook, NY 11794, USA; courtnae.alves@stonybrook.edu; 7 School of Social Welfare, Stony Brook University, Stony Brook, NY 11794, USA; kristina.cross@stonybrook.edu; 8 School of Social Welfare, University of Washington, Seattle, WA 98105, USA; ashliz@uw.edu; 9 College of Nursing, Villanova University, Villanova, PA 19085, USA; bridgette.brawner@villanova.edu

**Keywords:** HIV, drug use, STI, interventions, sexual health, Black girls

## Abstract

Background: The relationship between drug use and poor sexual health outcomes in Black adolescent females such as diagnoses of sexually transmitted infections, HIV, and early/unwanted pregnancy has been established in the literature. Yet, very few interventions have been successful in reducing the risk of poor sexual health outcomes and drug use for adolescent girls. Even more rare are interventions that are catered to specifically to Black girls in the United States, which is a group that has the highest rates of poorer sexual health outcomes and negative consequences associated with drug use. Therefore, this systematic review sought to identify and organize interventions that are focused on preventing HIV, STIs, early pregnancy and drug use for and include large samples of Black girls. Fifteen interventions were identified that met the review’s search criteria. Results: A total of 15 interventions that were published between 2005 and 2020 were included in the review. While all but one intervention focused on sexual health outcomes, two interventions infused drug use education for girls. Conclusion: This review provides recommendations for sexual health and drug use prevention researchers to engage in an intersectional framework and concludes with a summary of next steps to guide future research and policy work to address disparities that impact Black girls.

## 1. Background

Despite advances in HIV and sexual health programming in the United States, Black adolescent girls have the highest rates of sexually transmitted infections (STIs), early teen pregnancy rates, and are at greatest risk of contracting HIV in adulthood compared to White, Hispanic, and Asian adolescent girls [1]. As many new HIV and STI cases among adolescents result from sexual risk behaviors, many prevention interventions that are available focus solely on individual level behavioral change. Sexual risk behavior is defined in the literature as having many sexual partners in a specific time period, having condomless sex, and having sex while under the influence of drugs and alcohol [1]. Drug use and sexual risk behaviors have a close relationship as studies have indicated that drug use often precedes sexual risk behaviors such as drinking or using drugs while having sex [2]. While Black girls have historically had lower drug use rates compared to other ethnic minority girls, they face greater negative consequences such as lower fertility rates and reproductive health issues, engagement in sexual risk behaviors such as using drugs and alcohol while having sex, and reproductive health issues [3]. According to most recent statistics related to drug use among adolescent girls, drug use among Black girls is beginning to rise [4]. Factors including perceived racial discrimination, sexism, low self-esteem, and poor mental health symptoms may explain the sudden rise in substance use amongst Black girls [5,6,7]. As the rates of drug use begin to rise among Black adolescent girls, it is essential that prevention efforts become tailored to this group to address such factors [8] Most intervention research have addressed factors such as drug use, reproductive health, and HIV as separate issues. However, these factors are highly related and need a more comprehensive intervention approach. Due to the close relationship that sexual risk behaviors and drug use have in increasing changes of contracting STIs, HIV, and teenage pregnancy, and the disparities that Black adolescent girls are impacted by, it is essential that prevention interventions be targeted specifically to Black girls. However, to date, there is no comprehensive synthesis of prevention interventions for Black adolescent girls that address sexual health disparities (e.g., HIV, STI, and teen pregnancy) and drug use prevention interventions. Therefore, we synthesized the existing evidence from studies that were designed for Black adolescent girls and included a majority of Black adolescent girls.

## 2. Purpose of Study

Given the sexual health and drug use disparities that exist in the U.S., the purpose of this review was to assess sexual health and drug use prevention interventions that were available for Black adolescent girls. The objectives of this review were to (1) examine sexual health and drug use outcomes for interventions catered to and include large samples of Black adolescent girls and (2) identify best practices used in these interventions that effectively prevent drug use and improve sexual health outcomes.

## 3. Methods

**Figure 1:**
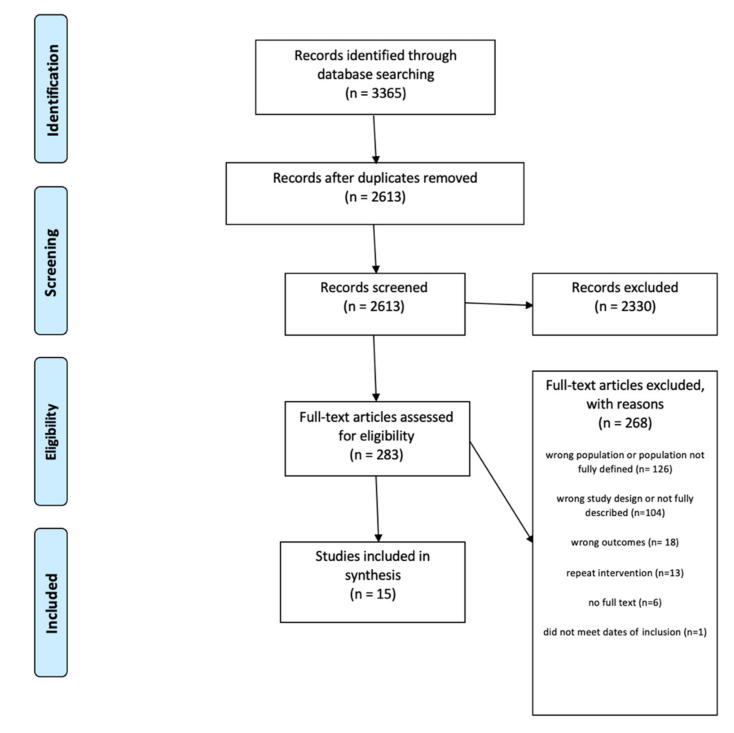
PRISMA.

The authors conducted a systematic review in accordance with the Preferred Reporting Items for Systematic Reviews and Meta-Analyses (PRISMA) guidelines (see Figure 1). The protocol was registered on 5 July 2020 in PROSPERO, an international database of prospectively registered systematic reviews. The registration number is CRD42020184201. Eligible studies met the following inclusion criteria: (1) interventions designed to reduce drug use, sexually transmitted disease (STI), and/or pregnancy (2) a majority of the participants were Black females between the ages of 11–19 (3) research was published in the United States between 2005 and 2020 in peer reviewed journals. The time range was selected due to only including the most recent and relevant interventions in the review.

## 4. Inclusion and Exclusion Criteria

To be included, the study had to include the following: (1) be a prevention intervention or quasi-experimental study designed to reduce drug use, improve HIV knowledge or sexual transmitted infections (STI) and/or prevent teen pregnancy; (2) a majority (over 50%) of the participants included must identify as Black females between the ages of 11–19; (3) studies have to conducted in the United States; (4) between 2005 and 2020 in peer reviewed journals; (5) have pre-post test results; (6) be in English only; (7) published in 2005–2020; and (8) be a peer-reviewed original study. The exclusion criteria were as follows: (1) duplicate studies using the same groups of participants; (2) study design that was not quasi-experimental or not randomized, or not considered a prevention intervention; (3) outcomes not pertaining to drug use or sexual health; (4) studies that do not provide results; (5) process manuscripts; (6) studies with no full-text available; (7) repeated intervention; (8) review articles; (9) did not have more than 50% of intervention participants that identified as Black adolescent girls; and (10) included samples of Black adolescents girls that were under the age of 11 or over the age of 19 years old.

## 5. Search Strategy

The following electronic databases were searched in April 2020 by a health sciences librarian who is one of the authors (L.S.): PubMed, PsycINFO, and Cumulative Index to Nursing and Allied Health Literature (CINAHL). In the search process, the intervention-related terms were used such as integrated, comprehensive, intervention, program, randomized controlled trial, and random controlled trial were combined using Boolean connector “OR”. The substance use related terms included: “Substance Use Disorders+/Policy & Control (PC)”) PC” OR “Alcohol Drinking+/Policy & Control (PC)”) OR “Smoking OR Tobacco OR Nicotine OR Smok* OR Cigarette OR Cocaine OR “Substance abuse” OR “ Substance use” OR “Drug use” OR “Drug abuse” OR Alcohol OR alcoholic OR Ethanol OR drinking OR marijuana OR Opioid OR Addict*. The terms related to sexual and reproductive health included: “HIV” OR “human immunodeficiency virus” OR AIDS OR “acquired immunodeficiency syndrome” OR “sexually transmitted” OR STI OR chlamydia OR syphilis OR gonorrhea OR herpes OR HPV OR “Human papillomavirus” OR “Sexually Transmitted Diseases OR “Pregnancy in Adolescence OR “Adolescent pregnancy” OR “teen pregnancy” OR “Unplanned pregnancy” OR “unwanted pregnancy” OR “unintended pregnancy”) OR “Adolescent pregnancy” OR “teen pregnancy” OR “Unplanned pregnancy” OR “unwanted pregnancy” OR “unintended pregnancy”. The terms related to race and gender included: “Blacks” AND “African American* OR Black*” AND “Female” OR Female* OR girl* AND (MH “Adolescence” OR “Minors OR Adolescen* OR teen* OR youth* OR student*. The results related to the type of intervention included: “Experimental Studies+” OR “Evaluation Research+) OR (MH “Program Evaluation”) OR Pretest AND posttest* OR program* OR evaluat* OR “effectiveness OR intervention” OR “clinical trial” OR “intervention”.

The search strategy combined key words and index terms related to the inclusion criteria and yielded a total of 3365 results. Results of the searches were imported into Covidence systematic review management software (Veritas Health Innovation Ltd., Melbourne, Australia) to facilitate the screening process. After duplicates were removed, the initial title and abstract screening of 2613 articles was completed by four reviewers (I.O., A.A., K.P., and L.S.) with two independent reviewers screening each record. Before this initial screening began, a sample of five records was screened by all reviewers and results were compared and discussed to enhance interrater reliability. Upon completion of title and abstract review, 2330 excluded because they did not meet the inclusion criteria. The full text review of 283 articles followed the initial screening and was completed by the same four reviewers with at least two reviewers again assessing each full text article to determine inclusion. The authors used the same criteria to review full text. After reviewing the full text articles, 268 manuscripts were excluded. Disagreements were resolved by the PI (I.O.). Fifteen studies were included in the final analysis (see Table 1). The last electronic search of data was on 15 May 2020.

## 6. Data Extraction

Four of the co-authors (I.O., K.P., M.A. and L.S.) extracted the following study-level data from records by using a common data collection spreadsheet, which include: Author, intervention name, location/setting, age/participant characteristics, type of study, components of session, eligibility/inclusion criteria, and study outcomes.

## 7. Quality of the Included Studies

The quality of the studies that were included in this review was assessed using the Joanna Briggs Institute (JBI) critical appraisal tools checklist [9]. For clinical trials, the JBI critical appraisal checklist for randomized controlled trials (13 criteria) was used. For quasi-experimental studies, the JBI critical appraisal checklist for quasi-experimental studies (nine criteria) was used. For cohort studies, the JBI critical appraisal checklist for cohort studies (11 criteria) was used. A score of more than 75% of the total indicated a high-quality study, whereas a score less than 75% of the total indicated a moderate- or low-quality study. Scores lower than low quality were not retained for the review.

## 8. Data Analysis

The data in this review were organized using a narrative synthesis approach [10]. Thematic analysis was undertaken in order to analyze the studies for the review. Thematic analysis was used in the process of narrative synthesis to develop codes and themes based on the selected intervention studies [11]. Themes emerging from the reviews based on outcomes related to the purpose of the review were extracted, together with a summary to illustrate each theme. The first author (I.O.) developed the initial codes and two authors (K.P. and M.A.) reviewed all codes to ensure that the codes reflected the data. Codes were then discussed, and any recommended changes were discussed and revised until full agreement was reached. After completing the thematic analysis, the codes of the intervention studies were merged and tabulated. The data were then translated into common categories/themes to allow for useful comparisons of the results and were discussed by the review team. All included studies were reviewed repeatedly to ensure the themes were representative.

## 9. Results

This study identified fifteen intervention studies that included Black adolescent girls solely or more than 50% of the participants were Black adolescent girls and addressed at least one or more of the targeted health outcomes commonly associated with sexual health, reproductive health, and drug use (see Table 1). The 15 interventions that were included in the review were all based in the U.S., in various cities and regions including: Atlanta, GA, Philadelphia, PA, and unspecified southeastern and northeastern cities. Interventions were delivered in multiple formats (e.g., CD-ROM, web-based, focus groups, session-format, discussions, and individual cell-phone interventions). Most interventions were conducted at community-based organizations or community clinics (*n* = 9), schools (*n* = 2), participant’s homes (*n* = 1), juvenile detention facility (*n* = 3), and web-based or computer (*n* = 2), and one television and radio based (*n* = 1). It is important to note that some interventions occurred in multiple locations (e.g., IMARA was conducted in both a juvenile detention center and then at the participants homes upon release). Eight studies within this review were randomized controlled trials [12,13,14,15,16,17,18,19] with a control group comprised of standard treatment or usual care, while two were randomized pilot studies with a control group [20,21]. Lawrence Zellner et al. [22] was not randomized although there was a treatment and control group. Three interventions had no control group [23,24,25]. Danielson et al. [26] was defined as a feasibility trial. The interventions were all catered to adolescents and primarily used HIV knowledge, STI knowledge, perception of risk, and daily drug use post intervention as main outcomes in their interventions. Two interventions used specimens to diagnose the presence of an STI using vaginal swab [15] and urine [14] of an STI post intervention as an outcome and pregnancy as an outcome [23].

## 10. Culturally Adapted Interventions

Cultural adaptations are defined as evidence based interventions that include elements, sessions, cultural beliefs, values, and language that are embedded in the target groups culture [27]. These elements can be infused within evidence based interventions to address a specific group [27]. Four interventions were specifically adapted for Black adolescent females [13,14,15,17], while three interventions were specifically catered for Black adolescents [18,22,25]. Four were specifically for adolescent females regardless of race [16,19,20,21]. Anderson et al. [24] was adapted for Black and Hispanic low-income youth although the study had a majority of Black female adolescents in the sample. The intervention was defined as culturally adapted if it included culturally specific materials or symbols (e.g., African proverbs or illustrations of Black people), if the intervention consulted with members of the target group, if the development of the intervention entailed use of cultural theory or existing Black literature, or if formative work was conducted with Black women [25]. Though the study was not considered culturally adapted and was not primarily catered to Black adolescent females, Key et al. [23] provided facilitators who identified as Black women to participants through the home visitation portion of the study. Authors of studies that were identified as culturally relevant or adaptive also emphasized the use of focus groups pre-intervention and advisory boards that comprised of Black adolescents to ensure that the intervention was culturally appropriate [22,24]. Intervention materials were aesthetically representative of the participants’ racial/ethnic diversity. Zellner et al. [22] infused culturally specific art forms, such as hip hop, West African dance, spoken word, and poetry, were incorporated into sessions to creatively reinforce prevention information and skills taught during the session [22]. While DiClemente [15] incorporate elements of Black feminist thought and the inclusion of Black women images within the interventions. Ethnic and racial identity and pride sessions were incorporated in five interventions [13,15,17,22,26] and measured as an outcome post intervention. Danielson et al. [26] and DiClemente et al. [13,15,17] both incorporated elements of Black feminist theory for their programs catered to Black girls while Miller et al. [25] incorporated Kwanzaa principles within each session.

## 11. HIV and STIs Prevention Interventions

Eleven interventions in our review focused specifically on STI/HIV education and prevention [13,14,15,16,17,18,20,22,24,25,26]. One of the interventions focused solely on HPV prevention and HPV vaccine awareness [13], while the remainder focused on STIs and HIV in general. The main components of interventions focused reducing HIV risk behaviors through education on STIs, HIV/AIDs knowledge, condom efficacy, and healthy relationships and sexual negotiation skills as the primary outcomes.

### 11.1. Increasing HIV and STI Knowledge

HIV and STI knowledge were a primary outcome in the majority of interventions included in this review [13,14,15,16,17,18,20,22,24,25,26]. The interventions were all able to demonstrate a significant increase in HIV and STI knowledge. For example, Miller et al. [25] found that at 16 week follow up, only 50% of its participants reported using a condom while having sex, although HIV and STI knowledge increased post intervention.

All HIV/STI prevention interventions in this review with the exception of the HPV vaccine awareness [13] focused on reducing behaviors associated with STI or HIV diagnoses including using condoms while having sex, reducing number of sexual partners as primary outcomes. The presence of an STI through biological assessments (e.g., STI testing in a clinic) was also used as a measure in three studies [15].

### 11.2. Self-Esteem and Efficacy

Among the interventions that focused on sexual health, seven of them incorporated sessions around self-efficacy and self-esteem [15,16,17,20,24,25,26]. Two interventions used incorporated motivational interviewing techniques in the study to target sexual risk behaviors [16,19]. Anderson et al. [24]’s Condom Carnival was the only intervention in our review to focused solely on condom usage, attitudes, and beliefs. While the study included both a sample of adolescent males and females, Black females in the study showed improvement in all outcomes such as lubricant safety. There was no other significant increase in participants’ perceived condom-related self-efficacy from pre-test to post-test. One study in our review focused solely on abstinence, Zellner-Lawrence et al. [22] which showed significant results in encouraged adolescent females to consider abstinence in the intervention group compared to the control group.

## 12. Drug Use as an Outcome

Only four interventions addressed drug use [12,14,19,21] as a specific outcome within their interventions that were either catered to Black adolescent females [14] or included a majority sample of Black adolescent females [12,19,21]. Wechsberg et al. [14] catered toward Black girls who were abusing drugs and led to significant reductions in marijuana use. Estefan et al.’s [12] “Dating Matters”, focused on teen dating violence, delinquency and drug use and used the adolescent substance involvement measure. The intervention reduced alcohol and drug use in adolescent girls relative to the comparison group. Mason et al. [21] used motivational interviewing using social networking strategies to reduce drug use and other risky behaviors. While this study focused on providing brief sessions that educated youth using social media on substance use education, the study found that for participants in the treatment, they were more likely to not use substance before having sex [21]. Other results include increased readiness to participate in mental health treatment and less trouble associated with alcohol and less access or offers to use marijuana after the treatment.

## 13. Teen Pregnancy

Only one intervention focused on pregnancy as the primary outcome [23], which was geared towards teen mothers to prevention subsequent unwanted pregnancies. The major components of this study included: intensive case management by a school-based social worker, through frequent home visits and continuous availability by cell phone; a weekly school-based peer educational and support group, with incorporation of group service learning; and comprehensive medical care for the teen mother and her child. Participants who were in the control group and received comprehensive medical case management were less likely to have a subsequent early pregnant than comparison group.

## 14. Discussion

Our systematic review of interventions for Black girls identifies the importance of interventions that target improving sexual health outcomes and preventing drug use. However, there is a critical need for the development of more interventions that are specifically targeted to Black girls and widely available. There are very few interventions that specifically target Black adolescent girls, while other interventions that focus on Black youth lacked a gender-specific focus. While many of the interventions included have several strengths, this review seeks to highlight those strengths while also challenging the field to incorporate important considerations that can advance the field further and provide necessary resources of Black girls as it pertains to their health.

### 14.1. School, Home, and Community-Based Interventions

Best practices for sexual health and drug use prevention intervention programs call for multi-layered, comprehensive, culturally competent approaches that encompass the intersectional identities and experiences of Black girls. The literature and research suggest that school-based interventions can positively impact sexual risk behaviors, HIV, STI outcomes, and unintended teen pregnancy for Black youth [22,23]. Among the interventions that were reviewed, three were school-based interventions. Zellner et al. [22] found that Black youth enrolled in school based sexual health programs reduced the likelihood of engaging in early sexual activity, HIV, STIs, and teen pregnancy. However, there are few interventions that are available for Black girls who are either not in school nor connected to schools or youth serving organizations. As school-based interventions may find it difficult to separate youth groups by gender and race, incorporating interventions either in home settings or online may be beneficial for Black adolescent girls to be inclusive. In addition, because the most marginalized groups of adolescents such as those in foster care are more likely to contract STIs, HIV, have unintended pregnancy, and engage in drug use [28,29,30], it is imperative that interventions are coordinated and promoted within local child welfare agencies to reduce the likelihood and vulnerability for youth in out-of-home care. Several studies have noted the efficacy and feasibility of implementing non-traditional, community-based or online sexual health, pregnancy, and drug use interventions. In particular, Mustanski et al. [31] found that implementing an online sexual health education program for LGBT youth had positive outcomes for participation, engagement, sexual health behavior outcomes. To date, there are no sexual health and drug use prevention interventions for Black adolescent girls who identify as LGBTQ. Interventions can be adapted to cater to specific sub-populations within Black adolescent girls. While limitations do arise and frequently occur when implementing online based intervention programs, such as lack of access to Internet, unknown intervention success, the outcomes of the studies posed thus far show a promising future for sexual health e-interventions.

Using non-traditional methods for intervention-based programming can be especially beneficial for people that historically have not reached out for sexual health care or health care in general. Another study more specific to adolescent girls of color, explored how a computer-delivered family intervention approach would prevent drug use among Black and Hispanic-American adolescent girls and successfully improved self-efficacy towards avoiding cigarette smoking, alcohol consumption and drug compared to the control group and experienced lower levels of depression [7]. While such prevention Interventions may exclude certain groups of Black girls that do not have access to the internet or cannot participate in a program in a private setting, their modes of interventions were deemed to be successful and innovative for Black girls whom were are able to access them. Mason et al. [21] provided students mobile phones to increase engagement and retention in the prevention intervention. Overall, incorporating interventions in non-traditional settings and mediums that allows for increased access to service utilization and education for people that have historically not engaged in prevention interventions conducted in traditional settings.

### 14.2. Infusing Sexual Health and Drug Use within Prevention Interventions

Only one intervention in our review included a focus on both on HIV prevention among Black girls who misuse drugs [14]. Previous research studies and literature have shown that drug use is a major risk factor for engaging in sexual risk behaviors [1,32]. For instance, both adolescents who use drugs socially and regularly are at increased risk of engaging in unprotected sex with multiple partners due to the influence of drugs and/or alcohol [33]. In addition, Black adolescent girls who drink experience higher rates of alcohol-related problems that includes illicit drug use, unprotected sex, and even higher rates of truancy which can lead to poor academic outcomes [34]. Studies have shown the benefits of drug use interventions on Black adolescent girls to reduce their engagement in sexual risk behaviors. For example, Lopez et al. [35], explored the benefit of a telemedicine prevention program for rural Black adolescent girls targeting drug use and sexual risk behaviors and found that Black adolescent girls gained skills in communication, safe sexual practices (condom use), decision making, and positive relationship building. This study was not included in our initial review as it was after our search was finalized. However, it is critical and essential that drug use interventions/strategies are integrated in HIV and sexual health interventions that are culturally competent and it is geared towards Black adolescent girls.

### 14.3. Inclusion of Biomedical Preventative Methods (e.g., PrEP and Black Adolescents Girls)

None of the studies reviewed included discussions of biomedical preventative methods. Though preexposure prophylaxis (PrEP) is highly effective in preventing HIV infection, the burden amongst Black youth is an area of concern [36,37]. Many contextual social and biological factors, including cognitive functioning, identity formation, experimentation, that place adolescents at an increased risk of contracting HIV [38]. While PrEP is a viable to HIV prevention, additional prevention methods, such as education, increase positive behavior change. Educating Black adolescent girls about PreP can lead to a sense of empowerment by arming them with available safeguards on preventing and reducing the possibility of HIV-related sexual risk.

### 14.4. Racism and Sexism as Risk Factors for Sexual Health Problems

A growing body of research has suggested that racism, sexism, exposure to discrimination increase sexual risk, likelihood of sexual risk behaviors, and contracting a STI [39,40,41,42]. There is a lack in discussion of any sexual health and drug use interventions that address racism and sexism for Black adolescent girls. The lack of empirical studies in drug use and sexual health prevention intervention that address, and incorporate issues of racism, sexism, and discrimination make it hard to pinpoint which intervention would be successful for Black girls. Specifically, among Black girls, few studies have revealed the connection between gendered racism, racial stereotypes and negative beliefs of self and its relationship with engaging in sexual risk behaviors. While interventions that are specifically catered to Black girls such as the original SIHLE/SISTA intervention [43] do incorporate sessions that highlight racial-ethnic identity and gender identity as a way to challenge negative view of self and increase confidence in Black girls, it is essential for interventions to incorporate more direct discussions about racism and sexism and the relationship on sexual health and drug use outcomes.

Intersectionality is a theoretical and epistemological framework that was promoted by Black feminist and critical race theorists and focuses on individuals intersecting identities and their connections to systems of oppression [42,43,44,45]. Intersectionality theory is rooted in Black feminist ontology and asserts that it is impossible and not justifiable to separate the racial, sexual, class, or gender-based oppressions because they are experienced simultaneously [46]. A need exists for interventionists to use intersectionality as a framework to address racism, sexism, and classism in sexual health and drug use prevention program models. The applications of intersectionality in sexual health and prevention intervention models would aim to understand the experiences at the axes of multiple oppressions, resist essentializing Black girls into a monolith, and commit to challenging the social injustices that Black girls often experience [47].

## 15. Conclusions

It is critical for public health interventions to provide safe spaces for Black adolescent girls to openly talk about their sexuality, drug use, and sexual risk behavior in order to reduce their risk of HIV/AIDS, STIs, early unwanted pregnancy, and drug use. This review focused on 15 years of sexual health and drug use interventions for Black adolescent girls. While many interventions are not catered specifically for Black adolescent girls, it is essential that interventions provided to Black adolescent girls incorporate the various intersections that Black adolescent girls belong to. The existing interventions were effective in addressing their primary sexual health or drug use related outcomes. This review, however, suggests that there is still a need to develop more integrated interventions that encompass both drug use and sexual behaviors as they often coincide to contribute to risk. In addition, we recommend the development of interventions that go beyond the individual level and address systemic racism and sexism which contribute to how Black adolescent girls view themselves and are treated in society. Further, there was a lack of sexual health interventions that focused on more comprehensive and holistic aspects of sexual health including sex positivity and pleasure. This illuminates the need for more holistic, asset-based sexual health interventions for Black adolescent girls to reduce sexual health disparities and drug use and promote positive well-being. We encourage researchers to incorporate various levels of strengths-based and culturally specific approaches and test the effectiveness of such interventions over longer periods of time.

## Figures and Tables

**Table 1:** Interventions that were included in the review.

Author’s Name	Intervention Title	Setting/Location	Theory	Age/Participants Characteristics	Type of Study	Components of Session	Study Outcomes	Findings
Anderson (2017)	Condom Carnival	Setting: Community-based organization Location: Large, southern, urban area, US	N/A	Mean age was 19.60 years, and the majority of the sample participants self-identified as Black.	Quasi experimental	Two peer facilitators per station (sessions). Six sessions involving condom knowledge, lubricants, and condom negotiation skills.	Lubricant safety knowledge, self-reported condom-related self-efficacy, and condom-related behavioral intentions and behavior likelihood.	Significant increase of participants who knew oil-based lubricants did not lower one’s chance of contracting HIV (*p* < 0.001). 97.8% of participants correctly answered a question about lubricant safety (pre = 82.2%). 13.2% of female participants improved significantly in lubricant safety awareness. A statistically significant increase in participants’ likelihood of carrying a condom in the next year, Z = −2.05, *p* = 0.04, with an effect size (r = 0.16).
Ito et al. (2008)	CD-ROM	Setting: Via Computer Location (recruitment): Wake County Health Department Adolescent, Family Planning Clinic, North Carolina, US	N/A	The average age of participants was 16 with a range from 15 to 19. 55% African American, 19% White, and 17% Hispanic.	Randomized controlled trial	Components of the CD-ROM intervention included: “Why do you care?”, “Know the facts”, “Protecting Yourself”, and “Sex and the media.”	Intended sexual behavior, HIV/STI knowledge, normative beliefs, attitudes, barriers, and self–efficacy regarding abstinence and condom use.	Intention to engage in sexual intercourse in the next 3 months decreased in both groups, nearly all participants intended to use a condom at next sexual intercourse, HIV/STI knowledge increased significantly in both groups, but there were no significant differences between the CD–ROM and comparison group in these factors. A difference in increased perceived barriers to condom use post intervention in the CD–ROM group versus the comparison group had a p value of 0.05.
Danielson (2013)	Sistas Informing Healing Living Empowering SiHLEWeb	Setting: Online Location (Recruitment): Large Southeastern city, USA	N/A	N = 41 African American girls aged 13 to 18 years (M = 15.85, SD: 1.42)	Quasi-experimental	Four, 1-h modules that simulates live group participation by using an interactive, video-based design to present Health Educator/Near Peer content, as well as follow five characters’ lives and development as they progress through the SiHLE program. Users could receive real-time feedback from video peers, Health Educator, and Near Peer.	Sexual behaviors and condom use, condom use self-efficacy, self-esteem, ethnic pride, partner communication, and knowledge change	Knowledge significantly improved for STD education and condom demonstration, t (25) = 3.46, *p* < 0.01. Condoms use self-efficacy significantly increased, t (16) = 2.41, *p* = 0.03.
Diclemente (2009)	HORIZONS	Setting: Community clinics Location: Atlanta, GA	Social Cognitive Theory, and the Theory of Gender and Power	African American adolescent females (N = 715), aged 15 to 21 years, seeking sexual health services.	2-Arm Randomized Controlled Trial	Two 4-h group sessions and 4 telephone contacts over a 12-month period, targeting personal, relational, socio-cultural, and structural factors associated with adolescents’ STD/HIV risk, and given vouchers facilitating male partners’ STD testing/treatment.	Biological: STI infection, Psychosocial: Knowledge of STD/HIV prevention was measured using an 11-item index. Condom use self-efficacy, Communication frequency with male partners about safer sex	Fewer adolescents had a chlamydial infection (42 vs 67; *p* = 0.04) or recurrent chlamydial infection (4 vs 14; *p* = 0.02). Girls reported a higher proportion of condom-protected sex acts 60 days preceding follow-up assessments (*p* < 0.001) and less frequent douching (*p* = 0.001). Girls were more likely to report consistent condom use in the 60 days preceding follow-up assessments (*p* = 0.01) and condom use at last intercourse (*p* = 0.005).
Diclemente (2014)	Imara	Setting: Juvenile detention facility Location: Atlanta, Georgia	N/A	All African American adolescent girls (between 13–17 years old (mean = 15.3 years of age)) N = 188.	Randomized controlled trial	Three individuals 90 min counseling intervention sessions conducted by a trained African American female health educator. The baseline session at the detention facility. The second session in the participants’ home s an average of 15 days after the first session. The third session was conducted in participants’ homes at 3-months post-randomization.	Condom use self-efficacy, HIV/STI knowledge.	Statistically significant differences were observed in the means between the intervention and control groups for condom use self-efficacy, HIV/STI knowledge, and condom use skills at both the 3- and 6-month assessments.
Diclemente (2015)	Girls OnGuard	Setting: Community clinic Location: Atlanta, GA	Information- Motivation- Behavioral (IMB) model	(N = 216) African American adolescent females were enrolled	Randomized control trial	One 12-min interactive computer-delivered media presentation on HPV vaccination. After the presentation, participants were given a motivational keychain to store a vaccine reminder card modeled in the video.	Intention to take the HPV vaccine	Approximately 12% of all study participants received the first dose of HPV vaccine, with an equal number of participants in the intervention and comparison conditions. The intervention group included more participants who completed the vaccine series (26 doses vs.17 doses in the comparison group)
Estefan (2020)	Dating Matters, Teen Dating Violence (TDV) Prevention Intervention	Setting: Schools Location not specific but recruited from four cities in USA	N/A	1750 females (53%), 1551 males (47%). The mean age was 11.93 years in the fall semester of 6th grade. Black, non-Hispanic (N = 1641, 50%) and Hispanic (N = 1022, 31%).	Clustered- randomized controlled trial	(1) classroom- delivered programs for youth in 6th, 7th, and 8th grades; (2) community-based parent programs for parents of 6th, 7th, and 8th grade youth; (3) a school-level intervention (educator training for all educators in schools receiving DM); (4) a “near-peer”-led youth communications program; (5) community-level activities to promote capacity and readiness assessment, policy development	Alcohol and drug use, delinquent behaviors, and weapon carrying	The average relative risk reduction in weapon carrying was 9%. The average relative risk reduction in alcohol and drug use was 9% with relative risk reductions between DM and SC ranging from 14 to 28%. The average relative risk reduction was 8% in delinquent behaviors with relative risk reductions between DM and SC ranging from 13 to 19%.
Gold (2016)	Computer-assisted Motivational Intervention (CAMI)	Setting: Community based organization Location: Pittsburgh, Pennsylvania	Transtheoretical Model and Motivational Interviewing	Participants were between the ages 13–21 years This study had a total of 572 female adolescents with a mean age of 17 years (SD = 2.2 years; range = 13–21 years; 59% African American)	A Longitudinal Randomized Controlled Trial	Three 30 to 45-min sessions of counseling at enrollment, and 3- and 6-month one-on-one brief counseling using MI with an interventionist guided by computer-generated feedback. Sessions included the fundamental principles of expression of empathy, development of discrepancy, ability to roll with resistance, and support of self-efficacy.	Self-reported sexual risk behavior, alcohol and drug use, unprotected sex frequency	Intervention reduced unprotected sex among an at-risk, predominantly minority sample of female adolescents. Due to the high attrition rate, the intent to treat analysis did not demonstrate a significant effect of the intervention on reducing the rate of unprotected sex.
Key (2008)	Intensive, school-based intervention	Setting: School Location not specified	Patient Centered Approach, Motivational Interviewing	99% African American teen (Average age is 16). 52% were currently pregnant and 47% were parenting	Prospective cohort design	The major components of the intervention were: (1) intensive case management by a school-based social worker, including frequent home visits and continuous availability by cell phone; (2) a weekly school-based peer educational/support group, with incorporation of group service learning; and (3) comprehensive medical care for the teen mother and her child, with coordination between the physician and social worker.	Subsequent births, contraception use	Subjects who participated in the comprehensive medical case components were more likely to use medroxyprogesterone (32/40, 80%) than those who did not participate (10/20, 50%) (*p* = 0.0145). The rate of subsequent births was lower in participants (17%) than in the comparison group (33%) (*p* = 0.001, hazard ratio = 2.5).
Zellner-Lawrence (2016)	2 HYPE Abstinence Club	Setting: Community based organization and juvenile detention center Location: Atlanta, Georgia, USA	Experiential Learning Theory, Social Learning Theory, Cognitive Learning Theory	The sample size *n* = 763 youth. 100% of the youth sampled identified themselves as Black/African American. Ages ranged from 12 to 18 years.	Randomized controlled trial	16 sessions which included West African art dance, poetry, communication skills, relationship building, and assertiveness skills	abstinence based measures, attitudes and beliefs about sex and marriage scale, behavioral intention to abstain from sexual intercourse	Participants were more likely to plan to be abstinent (odds ratio (OR) 1.41; 95% confidence interval (CI) 1.02, 1.95), males were two times more likely to plan abstinence compared to females (OR 2.00; 95% CI 1.45, 2.77). Participants who had not engaged in sexual activity were 2 times more likely to plan abstinence compared to participants that had been sexually active (OR 2.41; 95% CI 1.62, 3.60).
Mason (2011)	Motivational Interviewing integrated with Social Network Counseling Intervention	Setting: Community based organization Location: Philadelphia, PA	N/A	82% African American; 12% mixed race/ethnicity; 28 female adolescents; 14 completed the treatment intervention	Randomized control trial	20-min intervention organized into four component parts each lasting for 5 min: rapport building, presentation of substance use feedback from baseline assessment, introduction of social network information and presentation of feedback from baseline assessment, and summary and plans	substance use, sexual risk behavior, and mental health outcomes	Two substance use-related variables were significantly different, (a) using substances before sexual intercourse and (b) trouble due to alcohol use (p b 0.05), and both had large effect sizes, η2 = 0.18 and η2 = 0.15, respectively.
Miller (2017)	Becoming a Responsible Teen (BART)	Setting: Community based organization Location: Mid-west, United States	Information- Motivation- Behavioral Skills Model	African American Adolescents aged 14 to 18 years Participants mean age = 15.5 years, 52% female.	Quasi experimental	One session/week for 8 weeks with two community organizations	self-efficacy, HIV/AIDS knowledge, condom knowledge, condom intentions and attitudes	61% reported previous sexual intercourse. Adolescents demonstrated significant improvements in self-efficacy for safer sexual practices (*p* < 0.02), AIDS risk knowledge (*p* < 0.001), condom knowledge (*p* < 0.001), and condom attitudes (*p* < 0.04).
Redding (2015)	Step by Step	Setting: community-based organization and clinics Location: Metropolitan area of Philadelphia, Pennsylvania	Transtheoretical Model	Sample size was 828 14- to 17-year-old females. Race/ethnicity was mainly (84%) Black.	Randomized controlled trial	The computer randomized participants to either the TTM or SC group (1:1 ratio) within each recruitment site stratified by baseline stage of condom use. Participants completed the modular condom and smoking programs in 20–30 min and reports for both the participant and her counselor were printed.	smoking acquisition and cessation, sexual risk behavior, use of contraceptives while having sex	About 22% reported oral contraceptive use and 26% reported being pushed to have sex after refusal. The TTM group outcomes were significantly different from SC group outcomes for analysis of condom use at both 6 months and 12 months, but not at 18 months.
Sznitman (2011)	Project iMPPACS1	Setting (recruitment): Television and radio messages delivered over three years. Location: Syracuse, NY and Macon, GA	N/A	African American adolescents ages 14 to 17 N = 1710; (M = 15, SD = 1.05) with 763; 57% were African American females	Randomized controlled trial	Participants were randomly assigned to a small group HIV prevention or a general health intervention. Media messages were placed on TV shows popular to African Americans during the 16 month recruitment and 18 month intervention. Ads were replaced mid-way through the experiment.	condom negotiation, sexual risk behavior, condom beliefs, substance use, peer substance use	Reduced negative condom negotiation expectancies. Intervention group had a reduction in unprotected sexual activity.
Wechsberg (2017)	Young Women’s Co-op	Setting: Juvenile Detention Center Location: North Carolina	Empowerment Theory, African American/Black Feminist Theory	All Black female adolescents (between the ages of 16 to 19 years old) N = 118 Control group (N = 119)	Randomized controlled trial	Three individual content-driven sessions and one “dinner club” group session that supplemented the individual sessions and included circumstances unique to young adulthood. Including the importance of education, expectations of sex, information about teenage pregnancy, eating health food within a budget, etc.	Sex without a condom at last episode of vaginal intercourse, multiple sex partners, any marijuana use in the past 90 days, and heavy alcohol consumption in the past 90 days.	From baseline to 3 months follow up there was a statistically significant difference was found in the proportion of those in YWC who reported sex without a condom at last sex relative to the nutrition control (65% to 60% versus 46% to 61%), a significant reduction in any marijuana use (86% to 72%; *p* = 0.004) and for other risk behaviors, both study arms significantly decreased having multiple sex partners (37% to 22%, *p* = 0.005 for nutrition control; 39% to 28%, *p* = 0.05 for YWC).

## Data Availability

Data sharing is not applicable to this article as no datasets were generated or analyzed during the current study.

## Abbreviations

HIV	human immunodeficiency virus
STI	sexually transmitted infection
HPV	human papillomavirus
